# Molecular characterization of equine thymidine kinase 1 and preliminary evaluation of its suitability as a serum biomarker for equine lymphoma

**DOI:** 10.1186/s12860-021-00399-x

**Published:** 2021-12-14

**Authors:** Liya Wang, Lucia Unger, Hanan Sharif, Staffan Eriksson, Vinzenz Gerber, Henrik Rönnberg

**Affiliations:** 1 Department of Anatomy, Physiology and Biochemistry, Swedish University of Agricultural Sciences, Uppsala, Sweden; 2 Swiss Institute of Equine Medicine (ISME), Department of Clinical Veterinary Medicine, Vetsuisse Faculty, University of Bern, and Agroscope, Bern, Switzerland; 3 Alertix Veterinary Diagnostic AB, SE-392 30 Kalmar, Sweden; 4 Department of Clinical Science, Swedish University of Agricultural Sciences, Uppsala, Sweden

**Keywords:** Equine thymidine kinase 1, cancer, Serum biomarker, Equine lymphoma, Nucleoside analogues, Enzyme kinetics

## Abstract

**Background:**

Thymidine kinase 1 (TK1) plays a key role in the synthesis of deoxythymidine triphosphate (dTTP) and is thus important for DNA replication and cell proliferation. The expression of TK1 is highest during S-phase, and it is rapidly degraded after mitosis. In cancer cells, TK1 is upregulated, resulting in leakage of excess TK1 into the blood. Consequently, serum TK1 has been used as a diagnostic and prognostic cancer biomarker, mainly in human medicine. The aims of this work were to characterize equine TK1 and to evaluate its suitability as a serum biomarker for equine lymphoma.

**Results:**

Equine TK1 was cloned, expressed in *E. coli* and affinity purified. The purified recombinant horse TK1 showed broad substrate specificity, phosphorylating pyrimidine deoxyribo- and ribonucleosides and, to some extent, purine deoxynucleosides, including anticancer and antiviral nucleoside analogues. ATP was the preferred phosphate donor. Serum TK1 activity was measured in samples collected from horses with confirmed or suspected lymphoma and control horses with and without concurrent diseases. Serum TK1 activity levels were significantly higher in horses with lymphoma (*p* <  0.0005) and suspected lymphoma (*p* <  0.02) and in tumour-free groups with diverse diseases (*p* <  0.03) than in controls without concurrent diseases. There was a significant difference between the lymphoma group and the tumour-free group with diverse diseases (*p* <  0.0006). Furthermore, receiver operating characteristic analysis revealed a sensitivity of 0.86, a specificity of 0.95 and an AUC (area under the curve) of 0.92 compared to the controls without concurrent diseases, with a sensitivity of 0.97, a specificity of 0.71 and an AUC of 0.88 when compared with the tumour-free group with diverse diseases.

**Conclusion:**

Equine TK1 showed high specific activity and broader substrate specificity than human TK1. Anticancer and antiviral thymidine analogues were efficiently phosphorylated by horse TK1, suggesting that these analogues might be good candidates for chemotherapy in horses. Serum TK1 activity was significantly higher in horses with lymphoma than in controls. ROC analysis indicated that serum TK1 could serve as a promising cancer biomarker in horses.

**Supplementary Information:**

The online version contains supplementary material available at 10.1186/s12860-021-00399-x.

## Background

Like any other animals, horses can develop cancers. A recent survey of equine tumours in the United Kingdom found lymphoma to be the third most commonly diagnosed tumour after equine sarcoid and squamous cell carcinoma [1]. Lymphoma, although rare in the total equine population, is the most common haematopoietic neoplasm encountered in horses and can occur in horses of any age, with horses at 4–10 years most commonly affected [2]. There are five general types of lymphoma: multicentric, alimentary, mediastinal, cutaneous and solitary lymphoma. Multicentric lymphoma is the most common equine lymphoma type and shows a vast range of clinical presentations depending on which organs are affected. Alimentary lymphoma most frequently involves the small intestines, followed by the ascending and small colon, and causes malabsorption, which leads to severe weight loss, diarrhoea and sometimes colic [3]. Mediastinal lymphoma is characterized by neoplastic infiltration of the lymph nodes in the thoracic cavity causing compression of structures in the cranial mediastinum and thus leading to coughing, dyspnoea, distended jugular veins and muffled heart sounds secondary to thoracic effusion. Cutaneous lymphoma presents with multifocal, cutaneous nodules without internal metastasis, and solitary lymphomas may affect any organ, such as the spleen, or anatomical structures of the equine skull, such as the nasal passages or sinuses. Equine lymphoma is further classified according to its immunophenotype as of either B- or T-cell origin [2, 4, 5].

Clinical signs of cancer are generally vague and unspecific in horses and most commonly include apathy, weight loss, exercise intolerance and fever. In such cases, a systemic neoplastic disease should be considered as a differential diagnosis when more common causes, such as infectious diseases, severe parasite burden or dental or digestive disorders, have been ruled out, or if horses do not respond to standard therapy and show progressive loss of function of one or more organ systems [6]. Haematological and biochemical profile changes are also nonspecific and most importantly include anaemia, neutrophilia, hyperfibrinogenaemia, hypoalbuminemia and hyperglobulinemia [2]. Further diagnostic work-up may be quite extensive and invasive and often requires the expertise of a veterinary specialist. Depending on clinical signs, this work-up can include a rectal exam, abdominal and thoracic ultrasound, thoracic radiographs, abdomino- and thoracocentesis and fine needle aspirates or biopsies from internal organs collected with ultrasound guidance or during explorative laparotomy. The lack of reliable and noninvasive equine cancer biomarkers results in delayed diagnosis, ineffective treatment options and poor overall survival.

Deoxythymidine triphosphate (dTTP), one of the essential building blocks of DNA, is synthesized by two distinct pathways, e.g., the de novo pathway and the salvage pathway. In the salvage pathway, the synthesis of dTTP starts with the phosphorylation of thymidine catalysed by thymidine kinases, which is the rate-limiting step. Two thymidine kinases exist in mammalian cells: cytosolic thymidine kinase 1 (TK1) and mitochondrial thymidine kinase 2 (TK2). The expression of TK1 is cell cycle-specific, with its highest expression level during S-phase of the cell cycle [7], while the highest expression levels of TK2 are in the stationary phase [8]. The level of TK1 declines drastically after mitosis via a ubiquitin-proteasome regulated pathway [9]. In cancer cells, the cell cycle-regulated expression of TK1 is disrupted, resulting in overexpression and leakage of TK1 into the blood. Therefore, serum TK1 has been used in health screening to detect premalignant diseases and as a diagnostic and prognostic biomarker for cancer in human medicine [10, 11]. In veterinary medicine, serum TK1 has also been evaluated as a biomarker for diagnosis and prognosis, primarily in dogs with haematopoietic malignancies [12–14] but also in horses with lymphoma, with a sensitivity of 74% and a specificity of 86% and an AUC (are under the curve) of 0.80 as a diagnostic biomarker for this disease [15].

Pyrimidine nucleoside analogues such as AZT (azidothymidine) and 5FdU (5-fluoro-2′-deoxyuridine) have been used as chemotherapeutics for viral infection and cancer for more than 50 years. The activation of these nucleoside analogues by TK1 is a prerequisite for their antiviral and anticancer activity. In equine medicine, nucleoside analogues have been used for the treatment of viral infection and lymphoma [16–18]. However, equine TK1 has not yet been characterized; therefore, its role in the activation of therapeutic nucleosides is unknown.

The aims of this study were to characterize equine TK1 and to evaluate its serum level as a biomarker for equine lymphoma. Equine TK1 cDNA was identified and cloned into the pET-14b vector for expression in *E. coli*, and recombinant horse TK1 was affinity purified and characterized. Serum samples from healthy and diseased horses were collected, and the levels of TK1 were determined.

## Results

### Molecular characterization of equine TK1

#### Equine TK1 sequence analysis

**Fig. 1:**
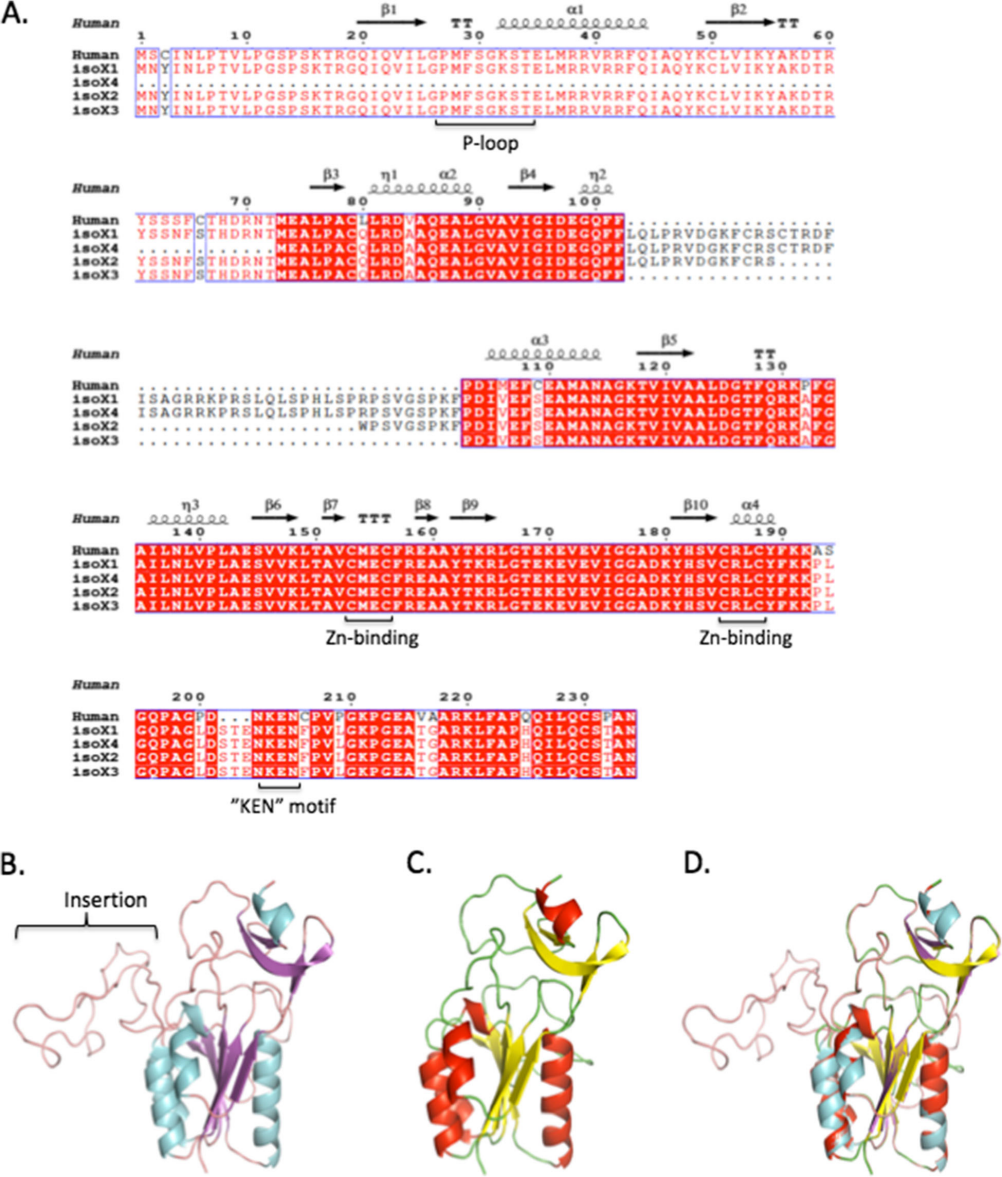
**A** Amino acid sequence alignment of equine TK1 isoforms with human TK1 using the Clustal Omega algorithm (https://www.ebi.ac.uk/Tools/msa/clustalo/) and structural alignment using ENDscript 2.0 software (http://endscript.ibcp.fr/ESPript/cgi-bin/ENDscript.cgi) with the human TK1 structure as the template. **B** Predicted 3D structure for isoform 1. **C** Predicted 3D structure for isoform 3. **D** Superimposed structures of isoforms 1 and 3. Structure prediction was performed with fully automated structural modelling software (https://sissmodel.expasy.org) using the human TK1 structure in complex with dTTP as a template (PDB code: 1W4R)

The horse TK1 gene (*tk1*) is approximately 12.6 kb long and is located on chromosome 11. At the mRNA level, there were four alternatively spliced transcripts found in the database, encoding proteins varying from 211 to 283 amino acids in length. However, which of the four transcripts is dominant is currently unknown. As shown in Fig. 1A, isoform 4 is the shortest transcript, and the predicted protein sequence lacks the ATP-binding loop (p-loop); thus, it is presumably nonfunctional. In isoforms 1, 2, and 3, all important functional motifs, e.g., the p-loop, the Zn-binding motif and the “KEN” motif, are present (Fig. 1A). However, an insertion of amino acid residues 46 (isoforms 1 and 4) and 22 (isoform 2) exists between α-helices η2 and α3 (Fig. 1A), which may form a large loop structure, as predicted based on the properties of the inserted amino acid sequence for isoform 1 (Fig. 1B) when compared with isoform 3 (Fig. 1C and D) using Swiss-Model, a fully automated protein structure homology server with the human TK1 structure in complex with dTTP as a template (PDB code: 1W4R). The formation of this loop structure may result in an inactive enzyme, since α-helices η2 and α3 play an important role in the formation of the active site structure, although in the predicted 3D structure of isoform 1, the loop structures are outside the core structure (Fig. 1B and D). Isoform 3 is the only transcript that codes for a polypeptide with primary and tertiary structures that are similar to those of human TK1 (Fig. 1A and C) and thus is considered the functional horse TK1. Horse TK1 (isoform 3) consists of 237 amino acids with a calculated molecular weight of 27.94 kDa and shows > 95% sequence homology to TK1 proteins in other species [19, 20].

**Fig. 2:**
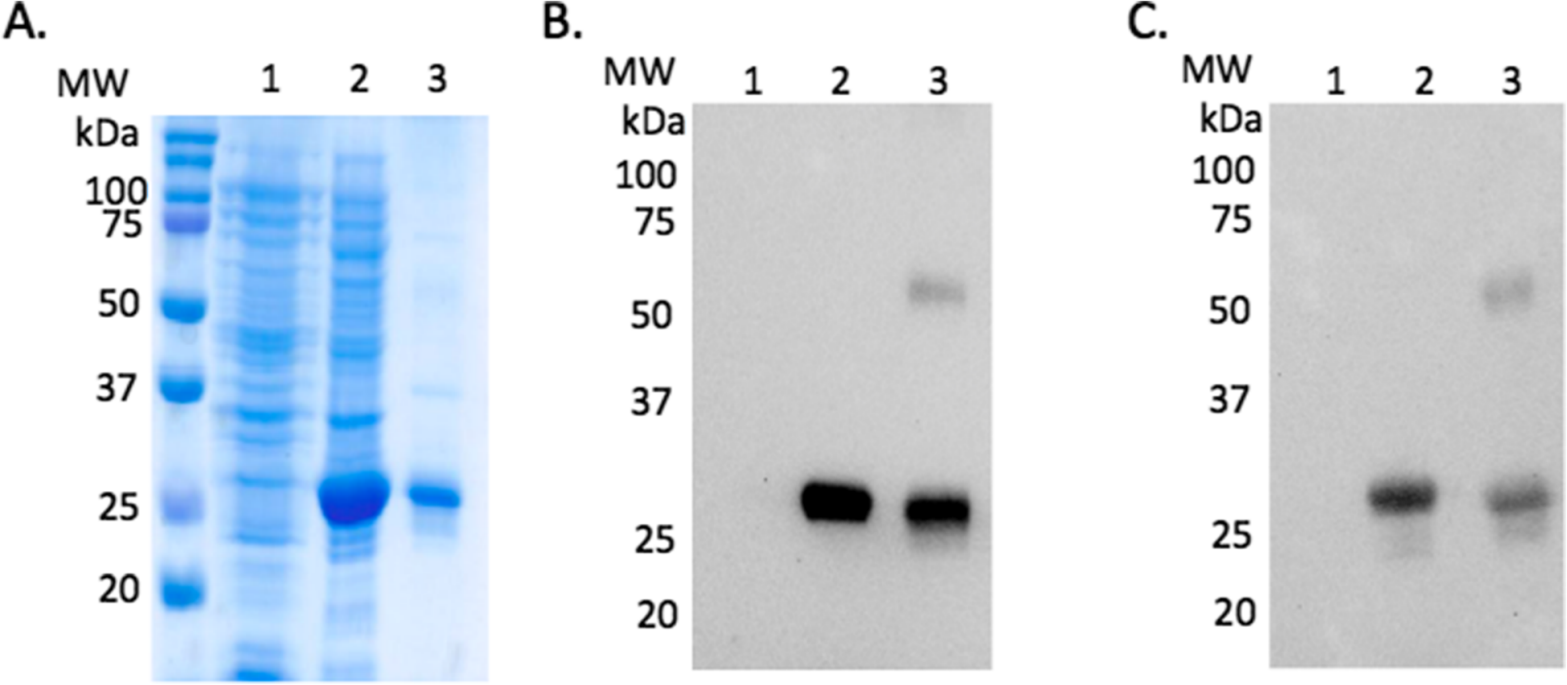
Characterization of recombinant horse TK1. SDS-PAGE analysis **A** and western blot analyses of recombinant horse TK1 with antibodies against 6xHistidine-tag **B** and TK1 **C**. Lane 1, *E. coli* extracts of uninduced culture; lane 2, *E. coli* extracts of induced culture; lane 3, purified recombinant horse TK1

To characterize the horse enzyme, isoform 3 cDNA was cloned into the pET-14b expression vector, and the recombinant horse TK1 protein was expressed in *E. coli* as a fusion protein with an N-terminal 6 X histidine tag to facilitate purification. The recombinant horse TK1 protein was then affinity purified to more than 95% purity by using Ni^2+^-Sepharose column chromatography (Fig. 2A). The yield of pure TK1 was ~ 2 mg per litre of culture. The identity of the purified recombinant horse TK1 was confirmed by western blot analyses using two specific antibodies, one recognizes specifically the 6xHistidine-tag (Fig. 2B) and one recognizes only the TK1 protein (Fig. 2C).

#### Substrate specificity

TK1 catalyses the transfer of a gamma phosphate group from a nucleoside triphosphate (phosphate donor) to the 5′-OH group of a nucleoside (phosphate acceptor), resulting in the formation of nucleoside 5′-monophosphate. Therefore, both phosphate acceptor and phosphate donor specificities were studied. Using ATP as the phosphate donor, a number of pyrimidine and purine nucleosides and some of their analogues were tested using a coupled spectrophotometric method. At a concentration of 100 μM, the activities of all the tested nucleosides were compared with that of thymidine (dThd, set to 100%). As shown in Table 1, among all the dThd analogues tested, TFT (trifluorothymidine) had higher activity, but AZT, FLT (fluorothymidine), and D4T (stavudine) showed lower relative activity than dThd. No activity was detected with α-dThd. Surprisingly, dUrd and its analogue 5FdU showed higher relative activity than dThd, while other tested dUrd analogues, e.g., L- and D-FMAU, FMAU, and FIAU, showed lower relative activity. Uridine (Urd) showed some activity, but not dCyd, cytidine, dGuo or dAdo (Table 1). These results demonstrated that horse TK1 has a broader substrate specificity than human TK1 [21].

**Table 1:** Substrate specificity

Substrate	Relative activity
dThd	100
Trifluorothymidine (TFT)	104.5
Azidothymidine (AZT)	55.0
Fluorothymidine (FLT)	43.5
α-dThd	< 0.01
Stavudine (D4T, 2′,3′.didehydro-2′,3′-dideoxythymidine)	6.4
dUrd	106.1
5-fluorodeoxyuridine (5FdU)	128.9
2′-deoxy-2′-fluoro-arabinofuranosyl-5-methyluracil (FMAU)	82.6
D-FMAU	96.2
L-FMAU	34.6
2′-deoxy-2′-fluoro-arabinofuranosyl-5-iodouracil (FIAU)	60.4
Uridine	1.7
Cytidine	< 0.01
dCyd	< 0.01
dAdo	< 0.01
dGuo	< 0.01

The assay was performed using a coupled spectrophotometric method at 21 °C. The substrate concentration was 0.1 mM with 1 mM ATP as the phosphate donor. The data are given as a percentage of that with dThd (0.95 μmol/mg/min)

Phosphate donor specificity was studied with 100 μM [^3^H]-dThd as the phosphate acceptor using a radiochemical assay. All natural nucleoside triphosphates at a 1 mM concentration could serve as phosphate donors, although dTTP only showed 0.1% relative activity. ATP showed the highest activity, while dATP showed ~ 71% relative activity, and other nucleoside triphosphates had ~ 12–24% relative activity compared with ATP (Table 2). Thus, as expected, the horse enzyme favours ATP as the phosphate donor.

**Table 2:** Phosphate donor specificity

Phosphate donor	Relative activity
ATP	100
UTP	19.1
GTP	23.9
CTP	13.7
dATP	70.7
dTTP	0.10
dGTP	13.9
dCTP	12.4

The assay was performed by using a radiochemical assay with [^3^H]-dThd as the substrate at 37 °C. The concentration of dThd was 0.1 mM, and the phosphate donor concentration was 1 mM. The data are given as a percentage of that with ATP as a phosphate donor (0.47 μmol/mg/min)

#### Steady-state kinetics

**Fig. 3:**
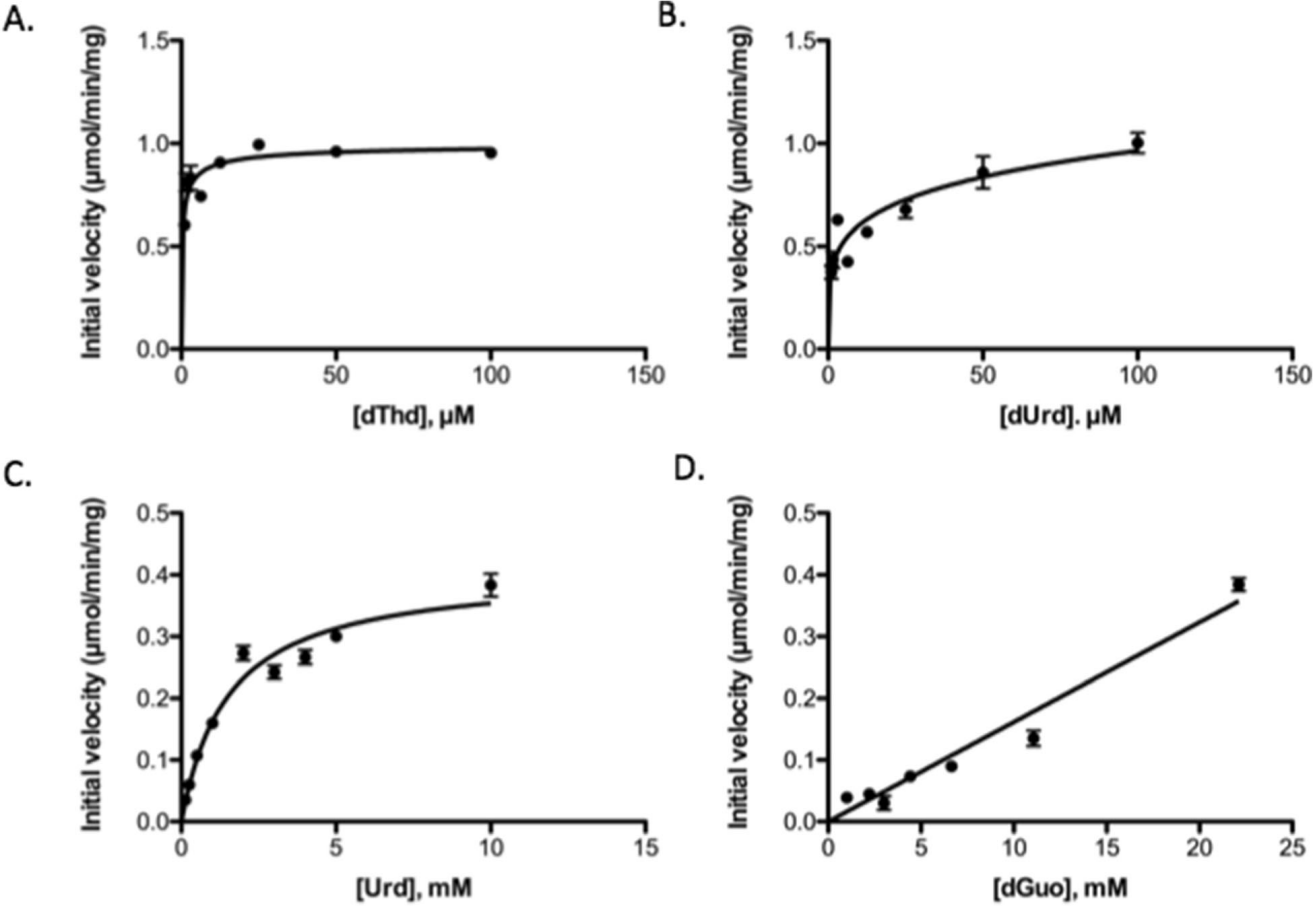
Steady-state kinetic analysis of horse TK1. Plots of initial velocity versus substrate concentration, dThd **A**, dUrd **B**, Urd **C** and dGuo **D**. The ATP concentration was kept at 1 mM

Steady-state kinetic analysis was performed using a coupled spectrophotometric method. As shown in Fig. 3A, the phosphorylation of dThd showed negative cooperativity with a Hill coefficient of 0.57 (Table 3). The values of the kinetic parameters K_M_ and V_max_ were 0.47 μM and 0.95 μmol/min/mg, respectively (Table 3). However, the phosphorylation of dUrd followed Michaelis-Menten kinetics (Fig. 3B), with K_M_ and V_max_ values of 1.64 μM and 0.81 μmol/min/mg, respectively. Due to the higher K_M_ value, the efficiency of dUrd phosphorylation was only 25% of that of dThd (Table 3).

**Table 3:** Kinetic parameters of horse TK1

Substrate	K_M_ (μM)	V_max_ (μmol/min/mg)	Efficiency (V_max_/K_M_)	Hill coefficient
dThd	0.47 ± 0.09	0.95 ± 0.02	2.02 (100)^a^	0.57
dUrd	1.64 ± 0.50	0.81 ± 0.05	0.49 (25.0)	1.0
5FdU	1.06 ± 0.2	1.08 ± 0.05	1.02 (50.4)	1.0
AZT	0.12 ± 0.08	0.53 ± 0.02	4.56 (225.9)	1.0
TFT	0.72 ± 0.28	0.93 ± 0.02	1.29 (64.0)	1.0
Urd	1511 ± 229	0.41 ± 0.02	0.00027 (0.013)	1.0
ATP	1501 ± 287	3.00 ± 0.29	0.0020	1.9

Kinetic parameters were determined using a coupled spectrophotometric method at 21 °C. To determine the kinetic parameters for phosphate acceptors, the ATP concentration was fixed at 1 mM, and to determine the kinetic parameters for ATP, the dThd concentration was fixed at 100 μM. The K_M_ and V_max_ values were calculated by fitting the initial velocity data into the Michaelis-Menten equation. The Hill coefficient was calculated by fitting the initial velocity data into the Hill equation

^a^Data in parentheses are relative efficiency compared with dThd (as 100%)

The kinetic parameters of selected antiviral and anticancer nucleoside analogues were also determined. The phosphorylation of 5FdU, TFT and AZT followed Michaelis-Menten kinetics, and the K_M_ values were 1.06 (5FdU) μM, 0.72 (TFT) μM, and 0.12 (AZT) μM, respectively, which are in the same range as dThd. However, the V_max_ values for these analogues varied; thus, their efficiencies were lower than that of dThd except for AZT, which had higher efficiency than dThd (Table 3). These findings are different from those for human and dog TK1 [20, 21].

The phosphorylation of Urd and dGuo was also investigated using a wider concentration range. As shown in Fig. 3C, Urd phosphorylation followed Michaelis-Menten kinetics with a K_M_ value of 1511 μM and a V_max_ value of 0.41 μmol/min/mg (Table 3), while the phosphorylation of dGuo did not reach the V_max_ value even at the highest concentration that could be achieved under these assay conditions; therefore, no kinetic parameters were calculated (Fig. 3D).

**Fig. 4:**
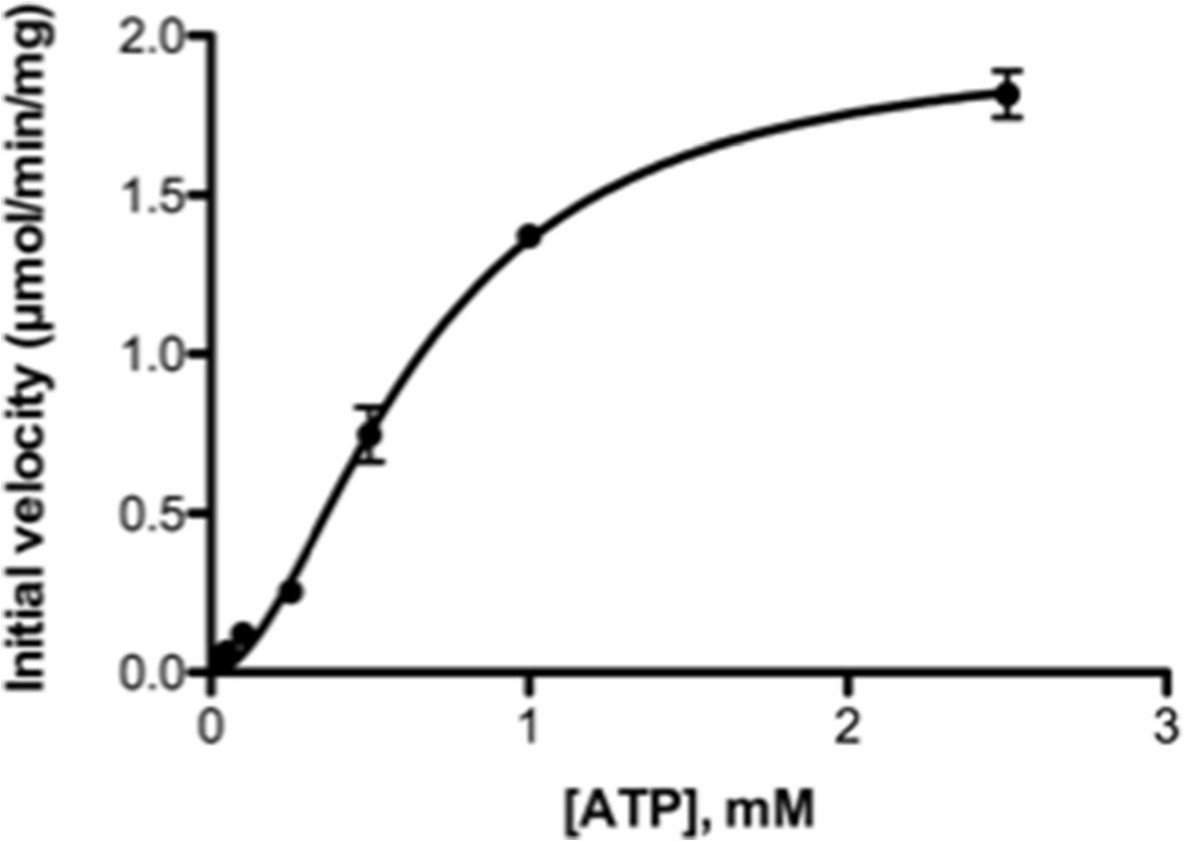
Steady-state kinetic analysis of horse TK1. ATP was the variable substrate, and the dThd concentration was kept at 0.1 mM

The kinetics of ATP showed positive cooperativity with a Hill coefficient of 1.9 (Fig. 4). The K_M_ and V_max_ values for ATP were 1501 μM and 3.00 μmol/min/mg, respectively (Table 3). These results suggest that the binding of ATP may affect subunit interactions, as observed in human TK1 [22].

#### Serum sample collection and serum TK1 analysis

**Fig. 5:**
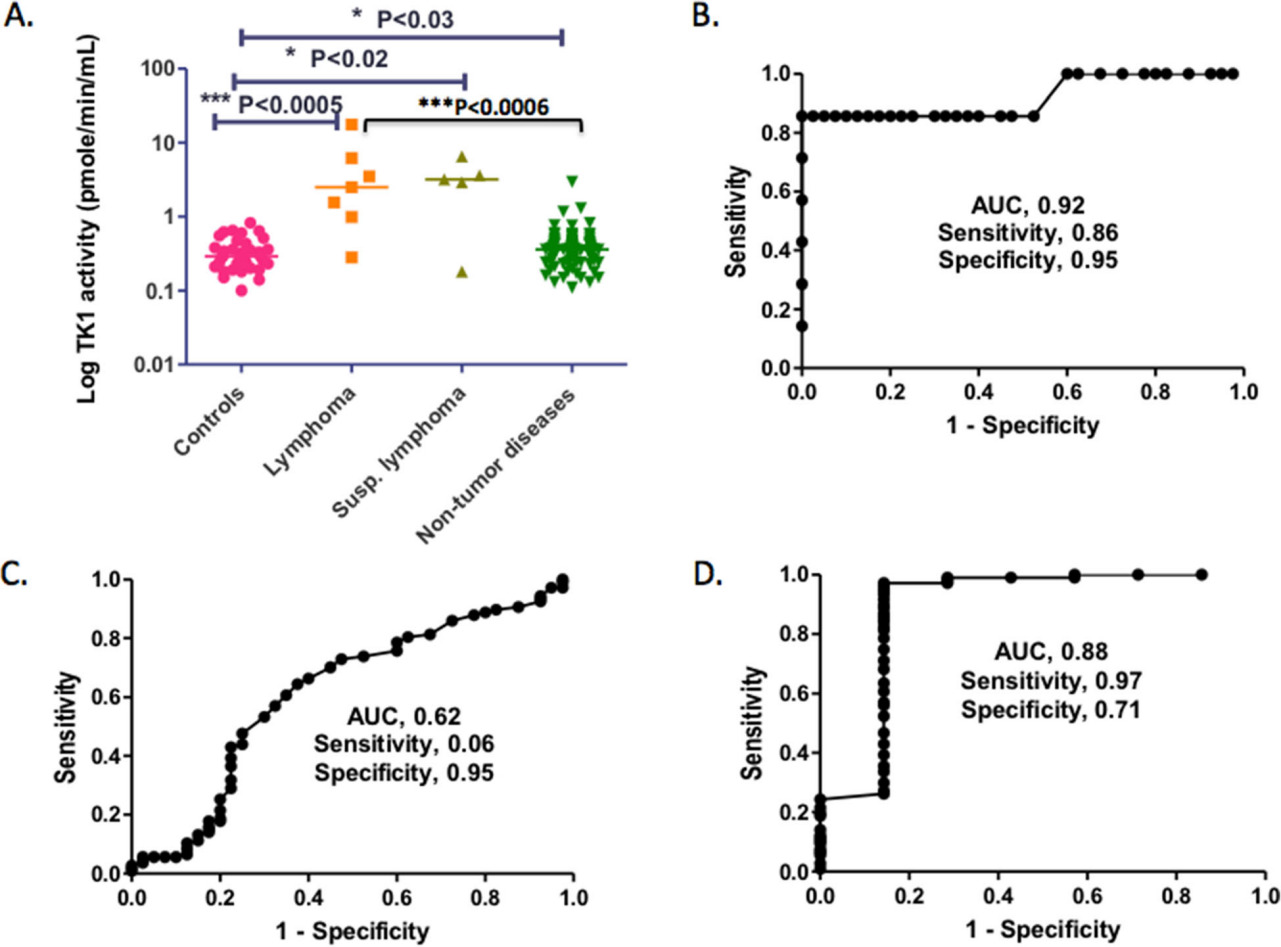
Analysis of serum TK1 levels. **A**. Comparison of serum TK1 levels in the control group without concurrent disease (Controls, *n* = 40), the lymphoma (n = 7) and suspected lymphoma (n = 5) groups and the tumour-free group with concurrent diseases (nonneoplastic diseases, n = 107). Bars represent the median. Receiver operating characteristic (ROC) analysis of serum TK1 activity to distinguish horses with lymphoma **B** and nonneoplastic diseases (nontumour diseases) **C** from the control group without concurrent diseases. The sensitivity and specificity were determined based on the chosen cut-off of 0.65 pmol/min/ml. To distinguish lymphoma from nonneoplastic diseases (nontumour disease group) **D**, the sensitivity and specificity were determined at a chosen cut-off value of 1.0 pmol/min/ml (mean + 2 SD)

Serum samples (including 1 plasma sample) were collected from 7 horses with confirmed lymphoma, 5 horses with suspected lymphoma, 107 control horses with concurrent diseases and 42 horses without concurrent diseases (detailed clinical information is provided in Supplementary Table 1). TK1 activity in serum samples was determined using [^3^H]-dThd as the substrate, a method developed previously for dog serum TK1 measurements and shown here to also work well for horse serum TK1 activity determinations [23]. The mean value and standard deviation and the median and range in each group are shown in Table 4. The mean serum TK1 activity of the control group without concurrent diseases was 0.33 pmol/min/ml with a standard deviation (SD) of 0.16 pmol/min/ml; thus, we could deduce a tentative cut-off value of 0.65 pmol/min/ml (mean + 2xSD). In the tumour-free control group with concurrent diseases, there were 5 out of 107 horses with serum TK1 activity above the cut-off value, and in the confirmed and suspected lymphoma groups, there was 1 horse in each group with serum TK1 activity below the cut-off value (Supplementary Table 1). As shown in Fig. 5A, the serum TK1 activity was significantly higher in the lymphoma (*p* <  0.0005), suspected lymphoma (*p* <  0.02) and tumour-free with concurrent diseases (*p* <  0.03) groups than in the controls without concurrent diseases, and there was a significant difference between the lymphoma group and the tumour-free group with concurrent diseases (*p* <  0.0006).

**Table 4:** Analysis of serum TK1 activity (pmol/min/ml)^a^

	Mean	Standard deviation	Median	Range
Controls (*n* = 40)	0.33	0.16	0.29	0.10–0.82
Confirmed lymphoma (*n* = 7)	4.7	6.1	2.5	0.28–17.7
Suspected lymphoma (*n* = 5)	3.3	2.2	3.2	0.18–6.5
Nontumour diseases (*n* = 107)	0.40	0.30	0.36	0.11–2.96

^a^Controls = horses without concurrent diseases (clinically healthy horses). Nontumour diseases = tumour-free horses with diverse concurrent diseases

ROC (receiver operating characteristic) analysis was conducted to evaluate the utility of serum TK1 as a biomarker and revealed a sensitivity of 0.86, a specificity of 0.95 and an AUC (area under the curve) of 0.92 for the confirmed lymphoma group using a cut-off of ≤0.65 pmol/min/ml. At the 95% confidence interval (CI), the AUC was 0.77–1.07 with a *p* value of 0.00045 when compared with the control group without concurrent diseases (Fig. 5B). For the nonneoplastic disease group (nontumour diseases), ROC analysis resulted in a sensitivity of 0.06, a specificity of 0.95 and an AUC of 0.62 using a cut-off value of 0.65 (Fig. 5C). ROC analysis of the lymphoma group against the nontumour disease group showed a specificity of 0.71, a sensitivity of 0.97 and an AUC of 0.88 using a cut-off of 1.0 pmol/min/ml, and at the 95% CI, the AUC was 0.7–1.1 with a p value of 0.0006 (Fig. 5D). These results demonstrated that serum TK1 could serve as a useful biomarker to distinguish individuals with lymphoma from control horses with and without concurrent diseases.

## Discussion

TK1 plays an important role in cellular dTTP synthesis, and its expression is closely correlated with DNA replication and cell proliferation. TK1 expression is also a prerequisite for the activation of certain thymidine analogues used in chemotherapy and as a diagnostic and prognostic biomarker for neoplastic disease. Therefore, it is necessary to characterize the molecular properties of equine TK1 enzyme in normal and neoplastic conditions to understand its potential role in veterinary medicine. In this study, we cloned, expressed and characterized equine TK1. Our results showed that horse TK1 preferred thymine and uracil as the base moieties of its substrate since dCyd, cytidine, dGuo and dAdo did not show any detectable activity under the same conditions. Modifications at the 5′-position of the thymine or uracil base resulted in higher activity. As expected, the enzyme preferred deoxyribose as its sugar moiety, as shown in Table 1. Urd had much lower activity than its corresponding deoxyribonucleoside, dUrd. Furthermore, modifications of the sugar moiety resulted in significantly decreased activity.

Horse TK1 is stringent regarding the configuration of its base since α-dThd showed no detectable activity, while the β-configuration (natural dThd) had 100% activity. The configuration of the sugar moiety also played a role in substrate selectivity since D-FMAU had 3 times the activity of L-FMAU and the activity of the racemic mixture FMAU was the average of those of the D- and L-form (Table 1). These results are consistent with what has been observed for human TK1 [24].

The results revealed that horse TK1 phosphorylated its natural substrate dThd with high efficiency, but the enzyme exhibited a somewhat broader substrate specificity than TK1 from humans and dogs [20, 21]. The kinetic behaviour of horse TK1 differed from those of human and dog TK1 in that all tested substrates followed Michaelis-Menten kinetics except dThd, which demonstrated negative cooperativity [20, 21]. The anti-HIV nucleoside analogue AZT showed higher efficiency than that of dThd due to its low K_M_ value. The anticancer nucleoside analogues 5FdU and TFT had > 50% relative efficiency compared with dThd. These results suggest that these analogues can be efficiently phosphorylated by horse TK1 and thus support further investigations of their potential use in antiviral and anticancer therapies.

Nucleoside analogues are widely used in antiviral and anticancer therapies; currently, > 50% of antiviral and anticancer drugs are nucleobases, nucleosides and nucleotide analogues. Nucleoside analogues are prodrugs that need to be activated by cellular nucleoside kinases to exert their therapeutic potential. The nucleoside analogues tested in this study, AZT, TFT, and 5FdU, are activated by TK1 and used for the treatment of HIV, herpes simplex virus type 1 and 2 and vaccinia virus infection or cancers in humans. This is of particular interest for the development of new treatment strategies for equine lymphoma, which has been recently associated with EHV-5 infection [25]. Nucleoside analogues such as cytosine arabinoside, acyclovir and valacyclovir have already been used as chemotherapeutic agents in horses with lymphoma. Both acyclovir and valacyclovir were associated with complete or partial remission in selected cases of EHV-5-associated lymphoma [17, 26, 27]. Although the antiviral and anticancer nucleoside analogues tested here have not yet been used in clinical settings in equine practice to date, the results presented here suggest that they may be good candidates for the treatment of cancers, viral infections or a combination of both in horses.

Biomarkers are molecules that can be objectively and easily measured and evaluated as indicators of normal and pathogenic processes and can be used in medical screening, diagnosis and treatment monitoring [28]. Serum TK1 activity has been used as a biomarker for health screening to detect premalignant diseases and for cancer diagnosis and prognosis in human medicine [11, 29]. In veterinary medicine, serum TK1 has also been shown to be a useful biomarker to diagnose malignant diseases in dogs and to monitor treatment [12–14, 20, 30]. Since equine lymphoma is a disease with many faces and usually unspecific symptoms, a reliable blood-based biomarker such as TK1 would greatly assist in the noninvasive identification of horses with lymphoma in not only advanced but also early stages of cancer. Indeed, in this study, we were able to show that the serum TK1 activity in horses with confirmed lymphoma was significantly higher than the activities in control horses with and without concurrent diseases. These results are in accordance with what was reported previously using a radioenzymatic technique (TK-REA assay) [15], but our assay method showed higher diagnostic sensitivity and specificity and a significantly higher AUC value upon ROC analysis. The test presented here was also superior to tests relying on serum IgM concentrations, which have a very poor sensitivity (23%) but a satisfactory specificity (88%) for diagnosing equine lymphoma at a cut-off of ≤23 mg/dl.

Our study cohort consisted of a relatively large and diverse group of control horses, including a population of old, well-monitored horses owned by a foundation, and thus offered a particularly adequate and reliable control group. As stated above, even though lymphoma is one of the more common forms of neoplasia in horses, in the general equine population it is nevertheless a rare condition limiting the number of confirmed cases referred to the ISME equine clinic throughout the sampling period to only seven. This might have decreased the statistical power and is the main limitation of this study. Additionally, we included a small cohort of horses with clinical signs highly suggestive of lymphoma, for which measurement of TK1 activity further corroborated this presumptive diagnosis. This shows that TK1 activity may be used as a diagnostic biomarker in daily equine practice when a more elaborate and invasive workup is denied by the horse owner.

The prognosis of lymphoma depends on the type and extent of metastasis; cutaneous lymphoma progresses slowly, and the affected horses may survive for several years with little or no treatment if no internal metastases occur. In contrast, for other types of lymphoma, such as generalized, alimentary or mediastinal lymphoma, the prognosis is poor, particularly if the disease is diagnosed at advanced stages. Therefore, early diagnosis is vital for the success of treatment and thus overall survival. Future studies evaluating serum TK1 as a diagnostic and prognostic biomarker for equine neoplastic conditions in larger study cohorts are warranted.

## Conclusion

Horse TK1 showed broader substrate specificity than TK1 from some other species, phosphorylating not only pyrimidine deoxyribonucleosides but also pyrimidine ribonucleosides and, to some extent, purine deoxynucleosides, including anticancer and antiviral nucleoside analogues. This information serves as a starting point for the understanding of the basic molecular biology of horse TK1 and its diagnostic and therapeutic uses in equine oncology.

The serum TK1 activity level in horses with malignant diseases was significantly higher than those of healthy horses and of horses with concurrent diseases, and there was no significant difference between the healthy and disease groups. These results suggest that serum TK1 can be a valuable biomarker to differentiate malignant from nonneoplastic diseases in horses.

## Material and methods

### Expression and purification of horse TK1

The horse TK1 gene was identified in the equine genome database, and cDNA encoding the horse TK1 protein was synthesized and cloned into the pET-14b vector (Genscript Inc.). The recombinant horse TK1 contains a 6X histidine tag and a thrombin cleavage site that are fused to the N-terminus of the horse TK1 sequence. Plasmid DNA containing horse TK1 cDNA was then transformed into the *E. coli* BL21 (DE3) pLysS strain, which was cultured in LB medium containing the appropriate antibiotics. The expression of the recombinant protein was induced by the addition of 0.1 mM IPTG to the culture medium, and recombinant horse TK1 was purified essentially as previously described [31]. Glycerol (10%) and dithiothreitol (DTT, 5 mM) were added to the final TK1 preparation, which was stored at − 70 °C in aliquots until further analysis. The purified horse TK1 was analysed by SDS-PAGE, and the protein concentration was determined using a Bio-Rad protein assay with bovine serum albumin as the standard.

### Western blot analysis

Aliquots of the *E. coli* cultures before and after induction with IPTG were centrifuged and the resulting pellets were resuspended in SDS-sample buffer and heated at 95 °C for 3 min to extract total proteins. These samples were then separated on 12% polyacrylamide gel together with the purified recombinant horse TK1. After electrophoresis the proteins were transferred to nitrocellulose membranes by semidry method. One membrane was probed with anti-6xHis antibody (GE-Healthcare) and the other was probed with anti-TK1 antibody (Alertix Veterinary Diagnostics, AB). The signals were detected by enhanced chemiluminescence (ECL) method (GE Healthcare) using Image studio system (Bio-Rad).

### TK1 activity determination and steady-state kinetics

Stock solutions of nucleosides and nucleoside analogues were prepared in DMSO and diluted in distilled water before use. The highest DMSO concentration in the reaction was 10%, which did not interfere with the TK1 activity measurement when tested with dThd as the substrate.

The initial velocities of the TK1-catalysed reaction with different substrates were determined using a coupled spectrophotometric method [32, 33]. Briefly, the reaction mixture contained 10 mM Tris/HCl, pH 7.6, 5 mM DTT, 5 mM MgCl_2_, 0.5 mM phosphoenolpyruvate, 0.1 mM NADH, 4 u/ml pyruvate kinase, 4 u/ml lactate dehydrogenase, 1 mM ATP, and variable concentrations of different substrates in a total of 0.5 ml. The reaction was started by the addition of purified horse TK1 (12 μg/ml), and the reduction in NADH concentration was monitored at 340 nm over time. The rate of product formation was equal to the rate of NADH oxidation in the reaction. All assays were repeated 3–6 times, and the results are given as the mean ± SD. The kinetic parameters were calculated by fitting the initial velocity data into the Michaelis-Menten equation, V_0_ = V_max_ [S]/(K_M_+[S]), or the Hill equation, V_0_ = V_max_ [S]^n^/(S_1/2_ + [S]^n^), where n is the Hill coefficient, and S_1/2_ is the substrate concentration required to reach ½ V_max_.

### Serum sample collection

Serum samples from horses with histologically confirmed lymphoma and suspected lymphoma were collected. The inclusion criteria for horses with suspected lymphoma were unexplained weight loss in combination with at least one further abnormal examination findings suggestive of lymphoma (abdominal and/or thoracic effusion, disturbance of anatomic function and structure of one or multiple organs, hypoalbuminemia, hyperfibrinogenemia, etc.) after exclusion of more common differential diagnoses such as infectious diseases, severe parasite burden or dental or digestive disorders. Furthermore, serum samples from control horses with or without concurrent diseases were collected in the population of horses referred to or owned by the ISME (Swiss Institute of Equine Medicine), University of Bern, Switzerland, and in a population of retired horses owned by a foundation in Switzerland. Horses were included as controls if they did not show clinical signs suggestive of a generalized, malignant internal neoplastic disease. Horses with PPID (pituitary pars intermedia dysfunction), also per se representing a neoplastic disease, were included since pituitary adenomas are considered benign and the prevalence of PPID is high in old horses [34]. Control horses were further categorized into two groups: control horses with and without concurrent diseases. Detail information of all control horses and horses with confirmed or suspected lymphoma is provided in supplementary Table 1. This project was approved by the Animal Experimentation Committees of the Cantons of Bern (BE110/15 and BE41/19+) and Jura (JU EXPE01/14+), Switzerland. All samples were collected with informed owner consent. All experiments were conducted in accordance with the ARRIVE guidelines (https:/arriveguidelines.org) and regulations presented by the Swedish Animal Protection Ethical Committee.

### Serum TK1 activity measurement

The TK1 activity in serum samples was determined using [^3^H]-dThd (PerkinElmer) as the substrate essentially as described [23]. Briefly, the reaction mixture containing 10 mM Tris–HCl pH 7.6, 2 mM DTT, 5 mM MgCl_2_, 5 mM NaF, 5 mM ATP, 5 μM [^3^H]-dThd, 10 mM NH_4_Cl and an appropriate amount of serum in a total volume of 40 μL was incubated at 37 °C for 60 min. Aliquots of the reaction mixture were spotted onto DEAE filter paper (DEAE filtermat, PerkinElmer) and dried. The filters were then washed 2 times in 1 mM ammonium formate. Thereafter, the filters were sorted, eluted in 0.5 ml buffer (0.1 M HCl and 0.2 M KCl) and counted in a scintillation counter (Tri-carb, PerkinElmer) after the addition of scintillation fluid (OptiSafe, PerkinElmer). All samples were assayed at least 3 times, and the results are presented as the mean ± SD.

### Statistical analysis

The distributions of serum TK1 activity levels in healthy and diseased groups were evaluated for normality using the D’Agostino and Pearson omnibus normality test. The serum TK1 activity levels showed non-Gaussian distributions, and the Mann-Whitney U test was used for comparisons between groups. Receiver operating characteristic (ROC) curves were constructed to evaluate the performance of horse serum TK1 activity. All statistical analyses were performed using GraphPad Prism 5.0 (GraphPad Software, La Jolla, CA, USA). Both ROC curves and median survival were calculated with the Kaplan-Meier curve using MedCalc version 17.6 (MedCalc software, Ostend, Belgium) statistical discovery software. A *P*-value < 0.05 was considered significant.

## Supplementary Information

**Additional file 1: Supplementary Table 1.** Overview of cases and controls.

**Additional file 2: Figure S1.** Original full length image of SDS-PAGE analysis shown in Fig. 2A. The SDS-gel was stained with Page Blue protein staining solution (Thermo Scientific) and distained with water. The wet gel was scanned directly. Half of the gel image is shown here because the other half is not relevant.

**Additional file 3: Figure S2.** Full length original image of western blot analyses shown in Fig. 2B & C. On the left is the original western blot image and on the right is a photo of the membrane taken after ECL detection with protein ladder seen. These image are directly exported from the software (Bio-Rad Image lab version 5.2.1) without any manipulation. The membrane was cut into two pieces after protein transfer, one was used for anti-his-tag antibody and the other one was used for anti-TK1 antibody. They were put together just before ECL detection. The membrane is outlined with black lines

## Data Availability

The authors confirm that all the raw data supporting the findings of this study either are presented in the article or can be found in the supplementary materials.

## Abbreviations

TK1: Thymidine kinase 1
dThd: 2′-Deoxythymidine
dUrd: 2′-Deoxyuridine
dCyd: 2′-Deoxycytidine
dAdo: 2′-Deoxyadenosine
dGuo: 2′-Deoxyguanosine
5FdU: 5-fluoro-2′-Deoxyuridine
TFT: Trifluorothymidine (5-trifluoromethyl-2′-deoxyuridine)
AZT: Azidothymidine (2′,3′-dideoxy-3′-azidothymidine)
FLT: Fluorothymidine (2′,3′-dideoxy-3′-fluorothymidine)
D4T: Stavudine (2′,3′-didehydro-2′,3′-dideoxythymidine)
FMAU: 2′-Deoxy-2′-fluoro-arabinofuranosyl-5-methyluracil
FIAU: 2′-Deoxy-2′-fluoro-arabinofuranosyl-5-iodouracil
Urd: Uridine
