# Supplementary material for: Molecular characterization of equine thymidine kinase 1 and preliminary evaluation of its suitability as a serum biomarker for equine lymphoma
Source: BMC Mol Cell Biol. 2021 Dec 14;22:59. doi: 10.1186/s12860-021-00399-x (PMC8670147; doi:10.1186/s12860-021-00399-x)
Supplement: Supplementary file 2 — Additional file 2: Figure S1. Original full length image of SDS-PAGE analysis shown in Fig. 2A. The SDS-gel was stained with Page Blue protein staining solution (Thermo Scientific) and distained with water. The wet gel was scanned directly. Half of the gel image is shown here because the other half is not relevant. [file 12860_2021_399_MOESM2_ESM.pptx]

## Slide 1
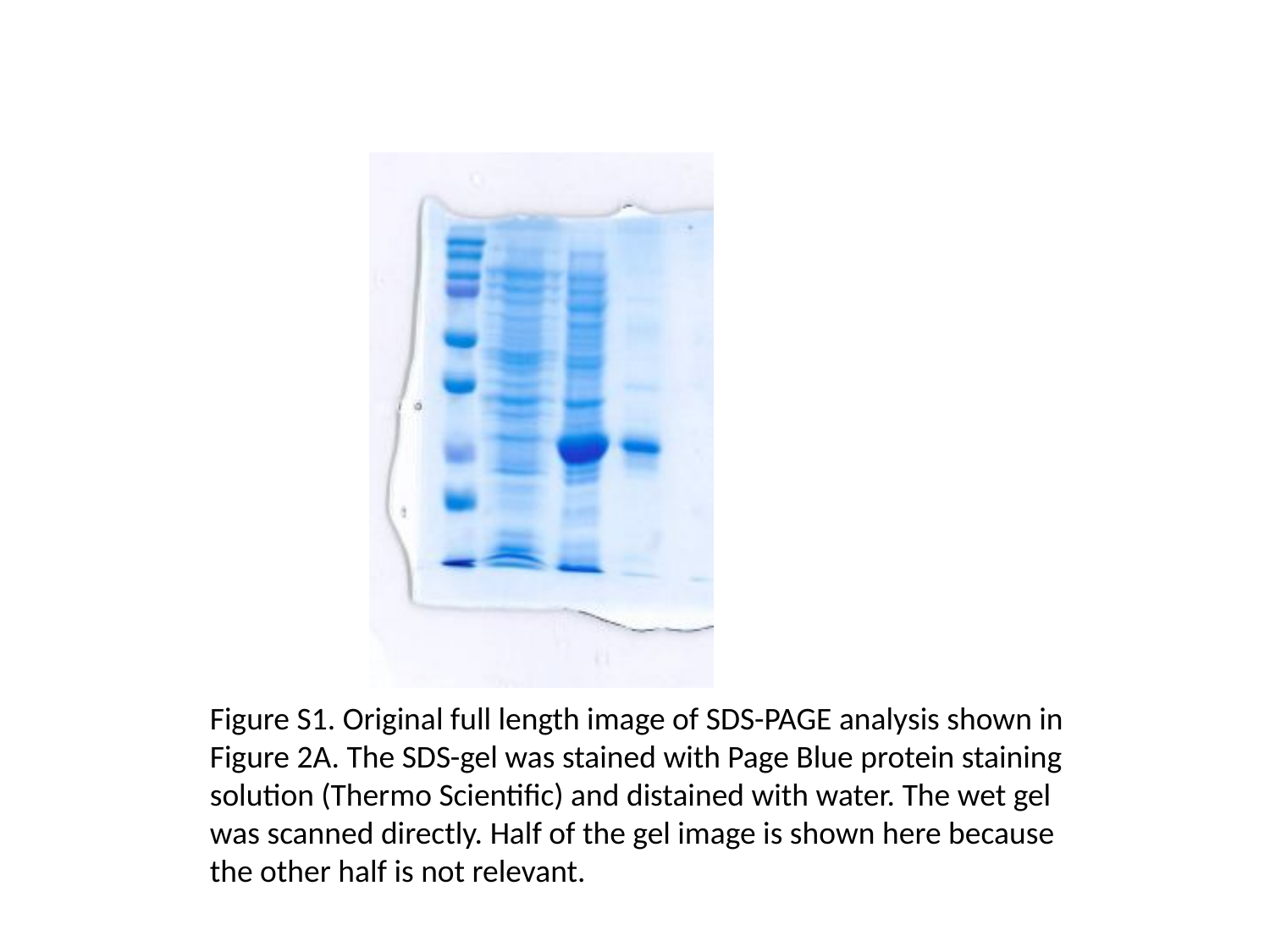

Figure S1. Original full length image of SDS-PAGE analysis shown in Figure 2A. The SDS-gel was stained with Page Blue protein staining solution (Thermo Scientific) and distained with water. The wet gel was scanned directly. Half of the gel image is shown here because the other half is not relevant.
