# Supplementary material for: Molecular characterization of equine thymidine kinase 1 and preliminary evaluation of its suitability as a serum biomarker for equine lymphoma
Source: BMC Mol Cell Biol. 2021 Dec 14;22:59. doi: 10.1186/s12860-021-00399-x (PMC8670147; doi:10.1186/s12860-021-00399-x)
Supplement: Supplementary file 3 — Additional file 3: Figure S2. Full length original image of western blot analyses shown in Fig. 2B & C. On the left is the original western blot image and on the right is a photo of the membrane taken after ECL detection with protein ladder seen. These image are directly exported from the software (Bio-Rad Image lab version 5.2.1) without any manipulation. The membrane was cut into two pieces after protein transfer, one was used for anti-his-tag antibody and the other one was used for anti-TK1 antibody. They were put together just before ECL detection. The membrane is outlined with black lines [file 12860_2021_399_MOESM3_ESM.pptx]

## Slide 1
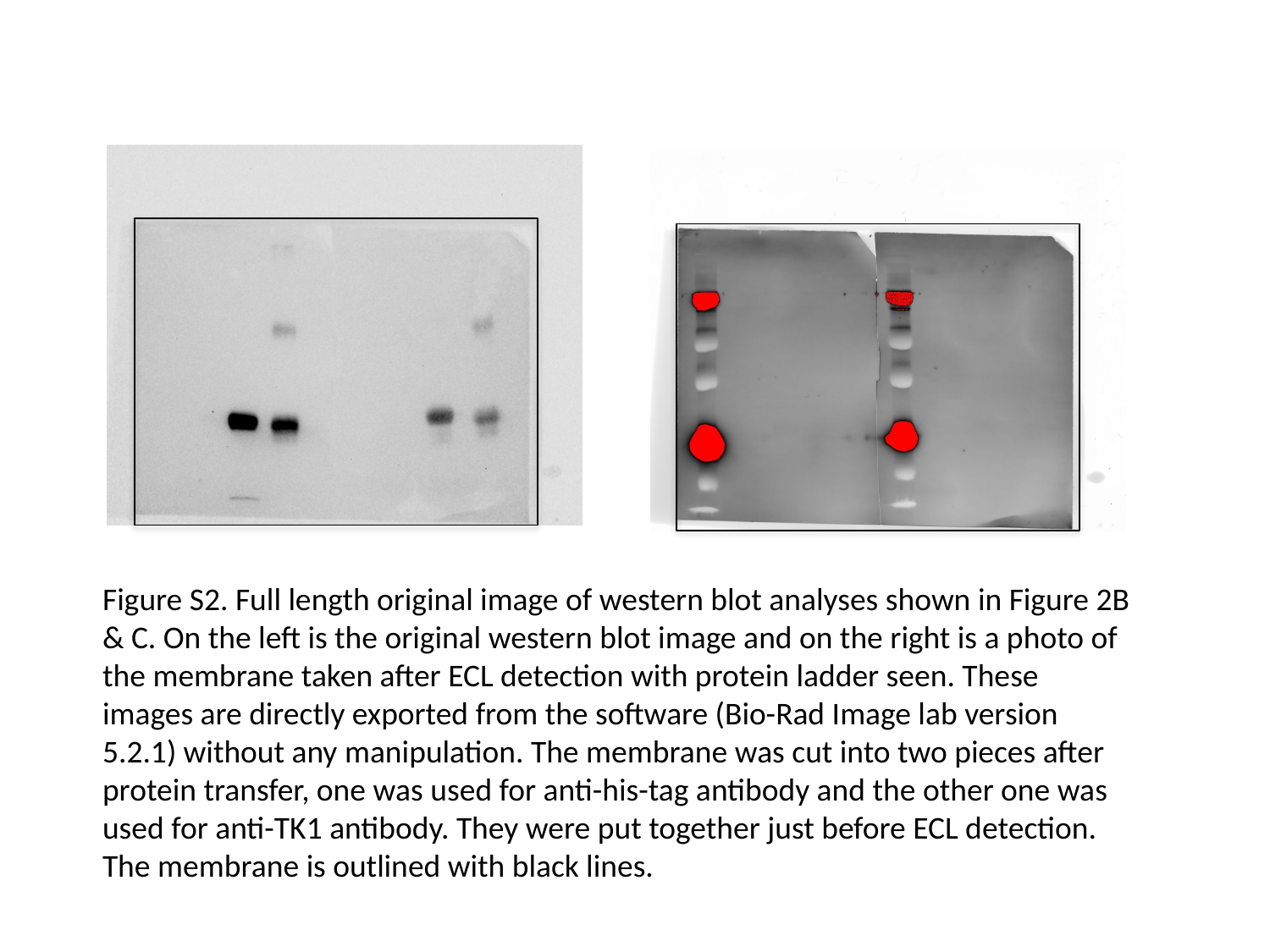

Figure S2. Full length original image of western blot analyses shown in Figure 2B & C. On the left is the original western blot image and on the right is a photo of the membrane taken after ECL detection with protein ladder seen. These images are directly exported from the software (Bio-Rad Image lab version 5.2.1) without any manipulation. The membrane was cut into two pieces after protein transfer, one was used for anti-his-tag antibody and the other one was used for anti-TK1 antibody. They were put together just before ECL detection. The membrane is outlined with black lines.
